# Nonoverweight nonalcoholic fatty liver disease and incident cardiovascular disease: A post hoc analysis of a cohort study

**DOI:** 10.1097/MD.0000000000006712

**Published:** 2017-05-05

**Authors:** Hashimoto Yoshitaka, Masahide Hamaguchi, Takao Kojima, Takuya Fukuda, Akihiro Ohbora, Michiaki Fukui

**Affiliations:** a Department of Endocrinology and Metabolism, Kyoto Prefectural University of Medicine, Graduate School of Medical Science; b Department of Diabetology, Kameoka Municipal Hospital, Kyoto; c Department of Gastroenterology, Murakami Memorial Hospital, Asahi University, Gifu, Japan.

**Keywords:** body mass index, cardiovascular disease, epidemiology, fatty liver disease, lean, NAFLD, obese

## Abstract

Nonalcoholic fatty liver disease (NAFLD) is known as a risk of incident cardiovascular disease (CVD). About 20% of NAFLD occurs in nonobese individuals. However, it remains to be elucidated the association between nonoverweight with NAFLD and a risk of incident CVD. Therefore, we investigated the risk of nonoverweight with NAFLD for incident CVD.

We performed a post-hoc analysis of the previous prospective cohort study, in which 1647 Japanese were enrolled. Abdominal ultrasonography was used to diagnose NAFLD. Overweight was defined as body mass index ≥23 kg/m^2^, which is recommended by World Health Organization for Asian. We divided participants into 4 phenotypes by existence of NAFLD and/or overweight. The hazard risks of the 4 phenotypes for incident CVD were calculated by Cox hazard model after adjusting for age, sex, smoking status, exercise, hypertension, hyperglycemia, hypertriglyceridemia, and low high-density lipoprotein cholesterol at baseline examination.

Incident proportions of CVD were 0.6% in nonoverweight without NAFLD, 8.8% in nonoverweight with NAFLD, 1.8% in overweight without NAFLD, and 3.3% in overweight with NAFLD. Compared with nonoverweight without NAFLD, the adjusted hazard ratios of incident CVD were 10.4 (95% confidence interval 2.61–44.0, *P* = .001) in nonoverweight with NAFLD, 1.96 (0.54–7.88, *P* = .31) in overweight without NAFLD, and 3.14 (0.84–13.2, *P* = .09) in overweight with NAFLD.

Nonoverweight with NAFLD was associated with higher risk of incident CVD. We should pay attention to NAFLD, even in nonoverweight individuals, to prevent further CVD events.

## 1 Introduction

Nonalcoholic fatty liver disease (NAFLD) has become an important public health issue because of its high prevalence.^[1]^ It has been reported that prevalence of NAFLD in general population is estimated to be 20% to 30% in Western countries and 5% to 18% in Asia, and it is increasing over time.^[2]^ NAFLD, which is now recognized as a component of metabolic syndrome,^[3,4]^ is known as not only a major cause of liver-related morbidity and mortality,^[5]^ but also a risk factor of cardiovascular disease (CVD).^[6]^ Recent studies revealed that NAFLD is associated with CVD through endothelial dysfunction.^[4,7]^

Nonalcoholic fatty liver disease has a close association with obesity, which is also known as a risk of incident CVD.^[8]^ Therefore, it has been believed that NAFLD individuals with obesity is a risk of incident CVD. In contrast, body weight of Asians is lower than that of the other races.^[9]^ In fact, about 20% of NAFLD occurs in nonobese individuals in Asian countries.^[10,11]^ We recently reported that nonoverweight individuals with NAFLD has higher diabetes risk than overweight individuals without NAFLD.^[11]^ In addition, recent studies revealed that normal-weight obese syndrome individuals who have normal body mass index (BMI), but have a high fat mass, are associated with chronic inflammation ^[12,13]^ and liver steatosis.^[4,14]^ Thus, NAFLD has an important role even in nonoverweight individuals. However, the risk of nonoverweight individuals with NAFLD for incident CVD remains to be elucidated.^[15]^ Therefore, we investigated whether nonoverweight individuals with NAFLD had an increased risk of CVD in this cohort study.

## 2 Materials and methods

### 2.1 Study population

To evaluate the impact of nonoverweight with NAFLD on incident CVD, we accessed a database from a previously reported study,^[6]^ which investigated the impact of NAFLD on incident CVD. Inclusion and exclusion criteria were described in the original publication.^[6]^ Briefly, we included the participants who received the checkups on an annual or biennial basis between January and December 1998. Exclusion criteria were current use of any medication, known liver disease, previous CVD event, or alcohol intake of more than 20 g/d. Participants who tested positive for hepatitis B antigen or hepatitis C antibody and those who reported a history of known liver disease, including viral, genetic, autoimmune, and drug-induced liver disease, were excluded as known liver disease.^[6]^

We interviewed between 2003 and 2004 by a self-administered questionnaire and investigated CVD events from 1998. If participants did not come back to the center during the follow-up periods, we sent the questionnaire by mail. We defined coronary heart disease, ischemic stroke, and cerebral hemorrhage as CVD. Coronary heart disease includes unstable angina, acute myocardial infarction, and silent myocardial infarction, and stable angina pectoris was not included. The participants who reported a doctor's diagnosis compatible with the above definition of CVD or those who reported signs and symptoms indicative of CVD were interviewed at the time of their visits or by phone. Thereby, we identified hospitals where the diagnosis of CVD was made and the diagnosis of CVD was confirmed by contact with the hospitals.

Habits regarding alcohol consumption were evaluated by asking the participants about the amount and type of alcoholic beverages consumed per week during the past month, and then estimating the mean ethanol intake per week.^[3,16]^ Participants reported the type, duration, and frequency of their participation in sports or recreational activities by the questionnaire.^[17]^ When participants performed any kind of sports at least once a week regularly, they were categorized as regular exercisers.^[3,16]^

The diagnosis of fatty liver was based on the results of abdominal ultrasonography.^[18]^ Of 4 known criteria (hepatorenal echo contrast, liver brightness, deep attenuation, and vascular blurring), the participants who had hepatorenal contrast and liver brightness were diagnosed as having fatty liver.^[18]^ Hypertension was defined as systolic blood pressure ≥130 mm Hg and/or diastolic blood pressure ≥85 mm Hg; hyperglycemia was defined as fasting plasma glucose ≥5.6 mmol/L; hypertriglyceridemia was defined as triglycerides ≥1.7 mmol/L; and low high-density cholesterol (HDL) cholesterol was defined as HDL cholesterol ≤0.9 mmol/L in men and ≤1.0 mmol/L in women.^[19]^ The Ethics Committee of Murakami Memorial Hospital approved the study and informed consent was obtained from all participants.

### 2.2 Definition of overweight and 4 phenotypes

Body mass index ≥23.0 kg/m^2^, which has been proposed as a cut-off for the diagnosis of overweight in Asian people,^[20]^ was defined as being overweight. This definition of overweight has often been used in Japanese population.^[11,21]^

Then, participants were categorized at the baseline examination into 4 phenotypes: nonoverweight individuals without NAFLD; nonoverweight individuals with NAFLD; overweight individuals without NAFLD; or overweight individuals with NAFLD.

### 2.3 Statistical analysis

The statistical analyses were performed using the JMP software version 10.0 software (SAS Institute Inc., Cary, NC) and *P* value <.05 was considered to represent a statistically significant difference. The study participants were divided into 4 groups based on the existence of fatty liver, and/or overweight and baseline characteristics of 4 phenotypes were compared. Continuous variables were expressed as mean ± SD, and categorical variables were expressed as number (percentage). The analyses of continuous and categorical variables to assess differences among 4 phenotypes were determined by Kruskal–Wallis test or Pearson chi-square test. Kaplan–Meier analysis was used for a graphical presentation of time to incident CVD, and log-rank test was used to assess difference among groups. To correct for family-wise error, we performed a Bonferroni correction, and a *P* value <.0083 was considered statistically significant in the log-rank test. Hazard ratio (HR) of the 4 phenotypes for incident CVD was calculated by Cox hazard model. The following variables were analyzed as potential covariates for the risk of incident CVD: age, sex, smoking status, exercise, hypertension, hyperglycemia, hypertriglyceridemia, and low HDL cholesterol at baseline examination.

## 3 Results

Between January and December 1998, 3835 participants had repeated the check-ups on an annual or biennial basis. Among them, 2429 participants (1680 men and 749 women) were agreed to include in the study. After exclusion, there were 1647 study participants (980 men and 667 women).

Baseline characteristics of participants according to 4 phenotypes were shown in Table 1. The proportion of nonoverweight participants with NAFLD was 22.1% among the participants with NAFLD. Participants with NAFLD had higher blood pressure, triglycerides, fasting plasma glucose, uric acid, aspartate aminotransferase (AST), alanine aminotransferase (ALT), and gamma-glutamyl-transferase (GGT), and lower HDL-cholesterol compared with participants without NAFLD among both nonoverweight and overweight participants. Among NAFLD individuals, overweight participants had higher blood pressure, fasting plasma glucose, AST, ALT, and GGT, and lower HDL-cholesterol compared with nonoverweight individuals.

**Table 1:**
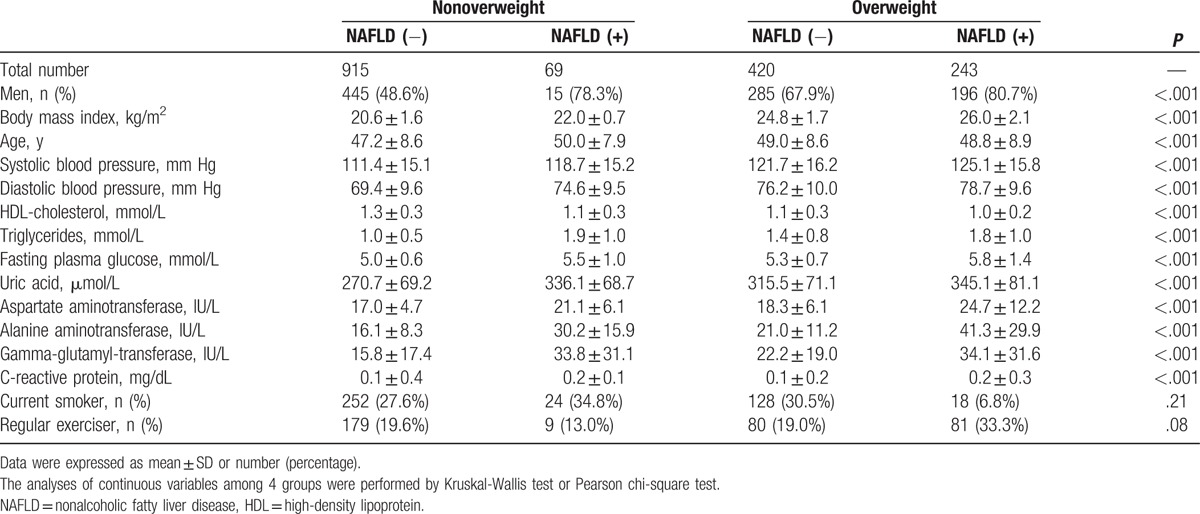
Baseline characteristics of participants.

**Figure 1:**
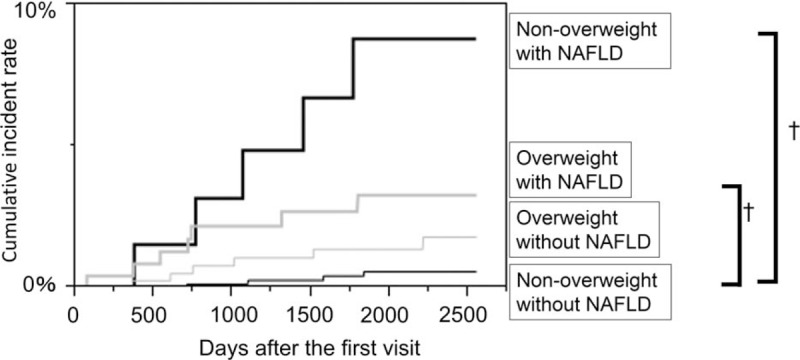
Hazard risk for the incidence cardiovascular diseases. The vertical axis is cumulative incidence of cardiovascular diseases and the horizontal axis is time as days. The black thin line represents nonoverweight without nonalcoholic fatty liver disease (NAFLD). The black bold line represents nonoverweight with NAFLD. The gray thin line represents overweight without NAFLD. The gray bold line represents overweight with NAFLD. The cumulative incidence rate of cardiovascular diseases among groups were evaluated by log-rank test. ^†^*P* value < .001.

Between January 2003 and December 2004, the incidence of cardiovascular events among the study participants was assessed by a self-administered questionnaire. We identified 22 events of CVD: 8 events of coronary heart disease (7 events of acute coronary syndrome and 1 event of silent myocardial infarction), 12 events of ischemic stroke, and 2 events of cerebral hemorrhage. The incident rate of CVD was higher in nonoverweight individuals with NAFLD (8.8%) than in other groups (0.6% in nonoverweight individuals without NAFLD, 1.8% in overweight individuals without NAFLD, and 3.3% in overweight individuals with NAFLD) (Fig. 1). The adjusted HRs for incident CVD compared with nonoverweight with NAFLD phenotype were as follows: 10.4 (95% confidence interval [CI] 2.61–44.0, *P* = .001) in nonoverweight individuals with NAFLD, 1.96 (95% CI 0.54–7.88, *P* = .31) in overweight individuals without NAFLD, and 3.14 (95% CI 0.84–13.2, *P* = .09) in overweight individuals with NAFLD (Table 2). In addition, compared with nonoverweight individuals with NAFLD phenotype, the adjusted HRs for incident CVD of overweight individuals without NAFLD and overweight individuals with NAFLD were 0.18 (95% CI 0.05–0.64, *P* = .01) and 0.32 (95% CI 0.10–1.08, *P* = .07), respectively. Thus, the risk of CVD of nonoverweight individuals with NAFLD was higher than that of overweight individuals without NAFLD.

**Table 2:**
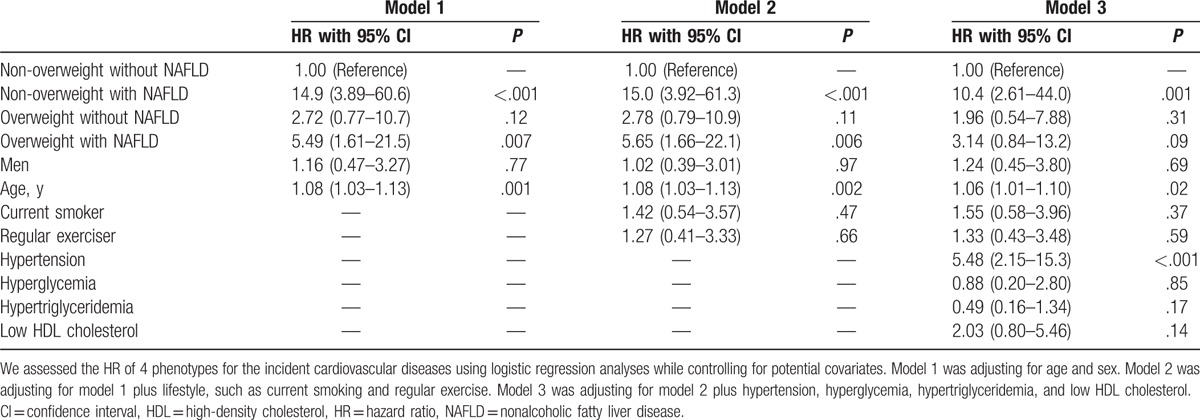
Hazard ratio of basic characteristics for the incident cardiovascular diseases.

## 4 Discussion

In this study, we showed that nonoverweight with NAFLD is associated with higher risk of incident CVD compared with nonoverweight without NAFLD or overweight without NAFLD. In addition, HR of incident CVD of nonoverweight with NAFLD was higher than that of overweight with NAFLD in our study population.

In this study, nonoverweight participants with NAFLD constituted a subgroup of individuals relatively free from the components of metabolic syndrome, which was in line with a previous study.^[22]^ Both NAFLD ^[6]^ and obesity ^[8]^ are known as risk of CVD. However, it has not been identified, which is more important as a risk for incident CVD, NAFLD, or overweight, because obesity is the dominant risk factor for NAFLD.^[3]^ We revealed that the risk of incident CVD of nonoverweight with NAFLD was higher than that of overweight without NAFLD. This result suggested that the existence of NAFLD had a more notable role on incident CVD than the existence of overweight.

Possible explanations for the association between nonoverweight with NAFLD and risk of CVD described as follows. NAFLD is caused by accumulation of ectopic fat in liver.^[23]^ It has been reported that epicardial fat, which is the ectopic fat in the heart, is associated with the incident CVD.^[24]^ Thus, the ectopic fat accumulation might be a key element for CVD, and hence the individuals with NAFLD is associated with the risk of incident CVD.^[6]^ Moreover, it was reported that individuals with NAFLD had a higher degree of insulin resistance compared with those without NAFLD, even if their fasting plasma glucose, fasting insulin levels, and serum lipids were within the normal range.^[25]^ Fat accumulation in liver induces hyperglycemia, dyslipidemia, and subclinical inflammation.^[26]^ In addition, it has been reported that the adipokines, such as adiponection, leptin, resistin, tumor necrosis factor (TNF)-α, and interleukin (IL)-6, which has close association with liver steatosis,^[27,28]^ is associated with CVD.^[29]^ Furthermore, Taylor and Holman^[30]^ hypothesized that ability to store fat is different among individuals and that ectopic fat increases easily in nonoverweight individuals, because nonoverweight individuals have low ability to store subcutaneous fat and visceral fat. Thus, nonoverweight individuals with NAFLD might represent the phenotype of low ability to store subcutaneous fat and visceral fat. Taking these findings together, the risk of incident CVD of nonoverweight with NAFLD is higher than that of the other phenotypes.

Recent studies show the concept of metabolically healthy obesity, which is characterized by low insulin resistance, is apparently protected from the metabolic complications of obesity.^[31]^ Because, there is an association between insulin resistance and NAFLD,^[3]^ the concept of obesity without NAFLD or nonobesity with NAFLD is similar to the concept of metabolically healthy obesity or metabolically abnormal nonobesity. Although the concept of metabolically healthy obese or metabolically abnormal nonobese phenotype is now well-recognized, however, there has been no unified definition of metabolically healthy or abnormal.^[32]^ In contrast, the definition of NAFLD is unified^[18]^; thus, it might be useful for using NAFLD as a marker of metabolic abnormality.

Some limitations of our study should be noted. First, fatty liver was diagnosed by ultrasonography. However, ultrasonography had a high sensitivity and specificity in diagnosing fatty liver.^[33]^ Second, our analysis is based on the incidence of self-reported CVD. There is a possibility that selection biases could have masked a true association between nonoverweight with NAFLD and CVD. Third, we could not assess insulin resistance, although insulin resistance would have an important role on incident CVD. Fourth, the data of diet regimen might affect the incident CVD. Unfortunately, however, we did not have data of diet regimen. Fifth, this is the retrospective post hoc study, and 12 years have passed since enrollment ended. Thus, gathering new information at a more recent time point is interesting to give more insights on the issue of our study. Unfortunately, however, we could not gather information at a more recent time point, because we already discarded the participants’ identification data. Finally, although the incidence rate of CVD in our study population was comparable with that of a much larger scale study reported recently from Japan,^[34]^ it was lower than those reported in Western countries, and so the generalizability of our study to non-Japanese populations is uncertain. In addition, the number of nonoverweight participants with NAFLD is relatively small. However, the proportion of nonoverweight participants with NAFLD was almost same as the past study.^[10]^

In conclusion, our study firstly showed an evidence that the risk of incident CVD in nonoverweight participants with NAFLD was higher than that in overweight participants. Thus, we should pay attention to NAFLD, even if they are nonoverweight, to prevent further CVD events. In addition, further investigations should be needed for elucidate the clinical features of nonoverweight with NAFLD.

## Acknowledgment

We thank all of the staff members in the medical health check-up center at Murakami Memorial Hospital.
